# Supplementary material for: Feld-induced modulation of two-dimensional electron gas at LaAlO3/SrTiO3 interface by polar distortion of LaAlO3
Source: Nat Commun. 2024 Jun 20;15:5268. doi: 10.1038/s41467-024-48946-2 (PMC11189907; doi:10.1038/s41467-024-48946-2)
Supplement: Supplementary file 1 — Supplementary Information [file 41467_2024_48946_MOESM1_ESM.pdf]

Supplementary Information

Field-induced modulation of two-dimensional electron gas at $LaAlO_3/SrTiO_3$ interface by polar distortion of $LaAlO_3$

Jinsol Seo, Hyungwoo Lee, Kitae Eom, Jinho Byun, Taewon Min, Jaekwang Lee,

Kyoungjun Lee, Chang-Beom Eom, Sang Ho Oh*

*E-mail: shoh@kentech.ac.kr (S.H.O)

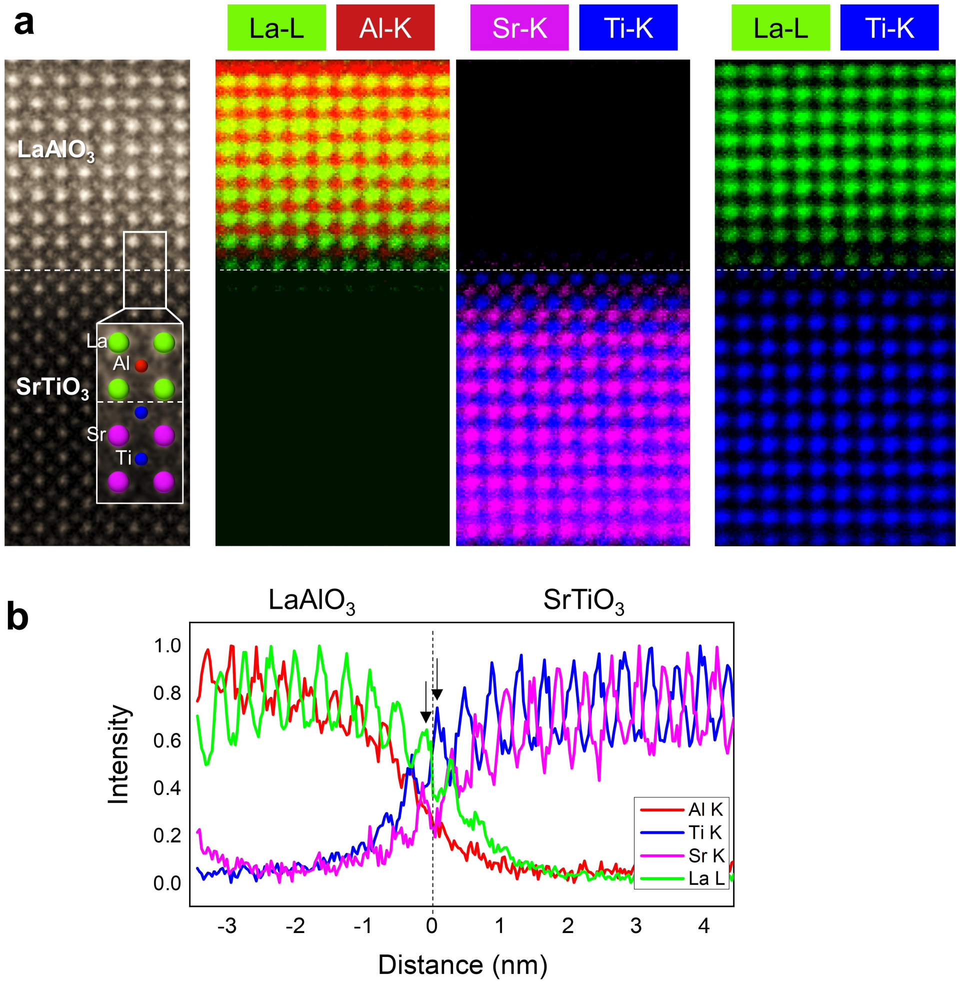

Supplementary Fig. S1. STEM-EDS analysis showing chemically abrupt *n*-type $LaAlO_3/SrTiO_3$ interface. a, STEM-HAADF image and EDS elemental maps prepared by collecting the characteristic X-ray of La-Lα and Al-Kα signals for $LaAlO_3$, and Sr-Lα and Ti-Kα signals for $SrTiO_3$, respectively. The composite map made of La-Lα and Ti-Kα clearly shows the LaO/TiO_2 interface termination. **b,** The intensity profile of each element across the interface. Arrows mark the TiO_2 termination of $SrTiO_3$ and LaO termination of $LaAlO_3$ at the interface.

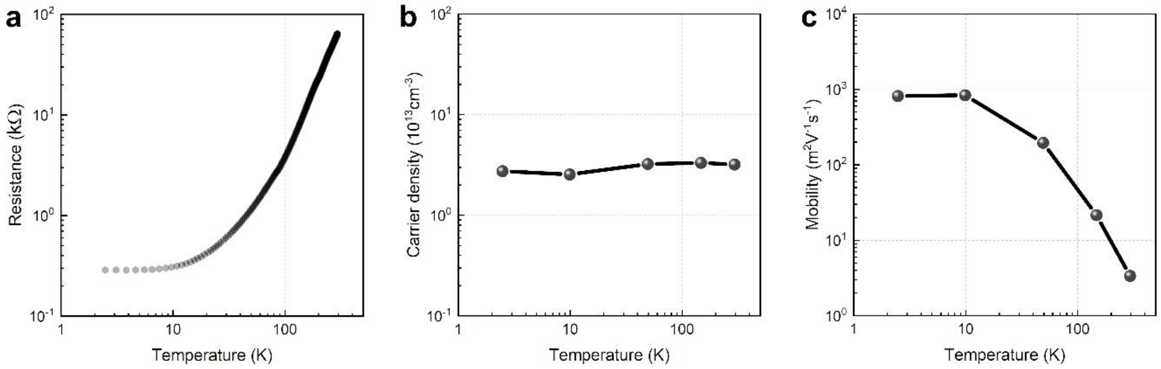

Supplementary Fig. S2. Transport measurement of a 10 unit cell thick $LaAlO_3$ grown on 15 unit cell thick $SrTiO_3$ (001) grown on $LaAlO_3$ buffered $SrTiO_3$ (001) substrate. The 10 u.c.-$LaAlO_3$/15 u.c.-$SrTiO_3$/3 u.c.-$LaAlO_3$ model structure was grown on undoped $SrTiO_3$ (001) substrate without $SrRuO_3$ top electrode for the electrical transport measurement. (a) Resistance, (b) carrier density, and (c) mobility.

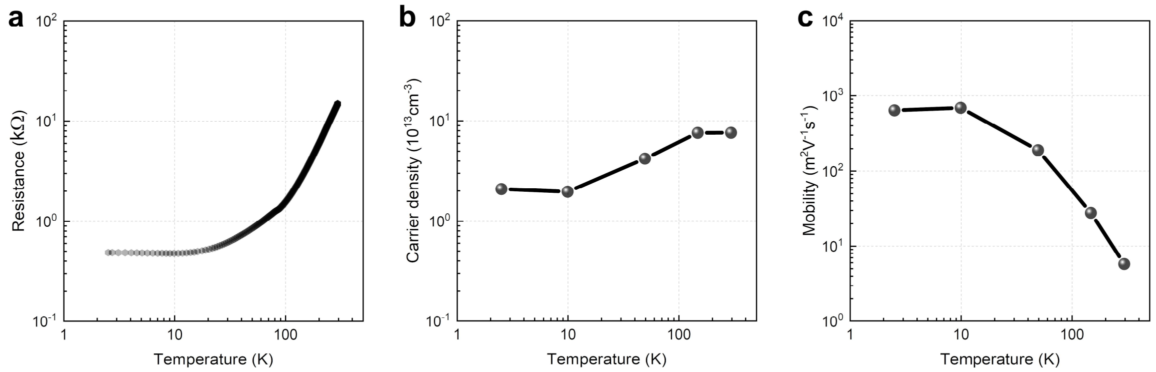

Supplementary Fig. S3. Transport measurement of a standard 10 unit cell thick $LaAlO_3$ grown on a $SrTiO_3$ (001) substrate. (a) Resistance, (b) carrier density, and (c) mobility.

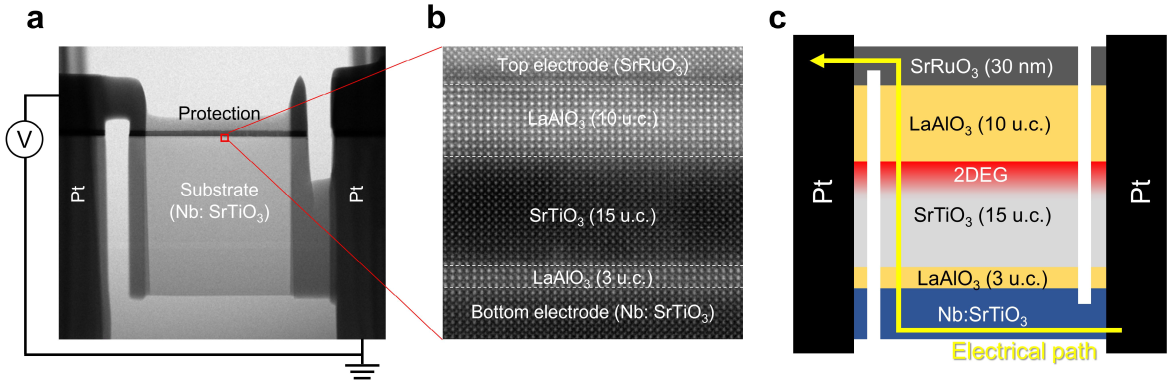

Supplementary Fig. S4. TEM sample prepared for the in-situ electrical biasing experiments. a, Low-magnification TEM image of the specimen. The TEM lamella was prepared by using FIB and attached to a MEMS-based chip designed for in-situ biasing experiments. **b,** HAADF STEM image showing the $LaAlO_3/SrTiO_3$ heterostructure device. **c,** Schematic drawing showing how the top ($SrRuO_3$) and bottom ($Nb:SrTiO_3$) electrodes are connected to the external metal (Pt) pads. The electrical path is indicated by the yellow line.

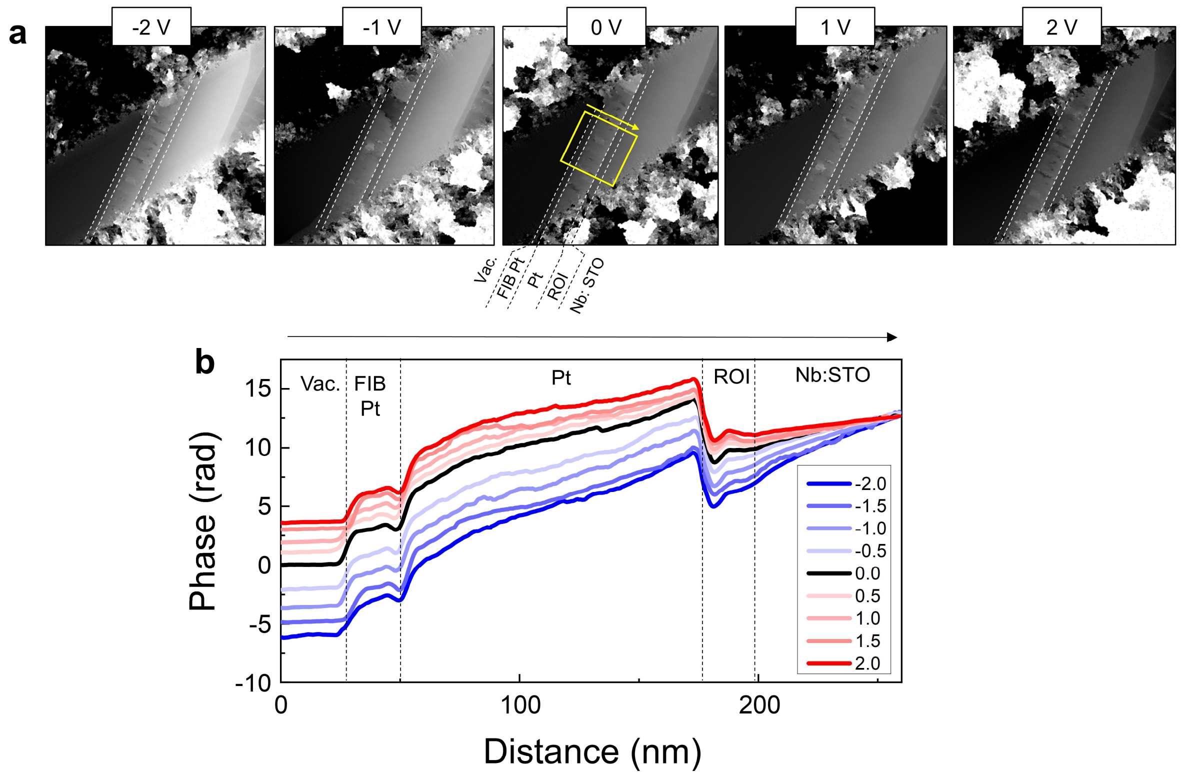

Supplementary Fig. S5. Potential mapping across the electrodes under the applied voltage using off-axis electron holography. a, Phase images of the $LaAlO_3/SrTiO_3$ heterostructure device with the voltage (from -2 V to +2 V in 1 V step) being applied to the $SrRuO_3$ top electrode. Off-axis electron holography has been used to reconstruct the phase of the electron beam. The region where the phase profiles were obtained is indicated by yellow line. **b,** Line profiles of the phase sampled from the region indicated in **a**. The phase shift, initiated at the electrically grounded electrode (Nb:$SrTiO_3$) substrate, increases linearly with the applied voltage and remains constant in the vacuum.

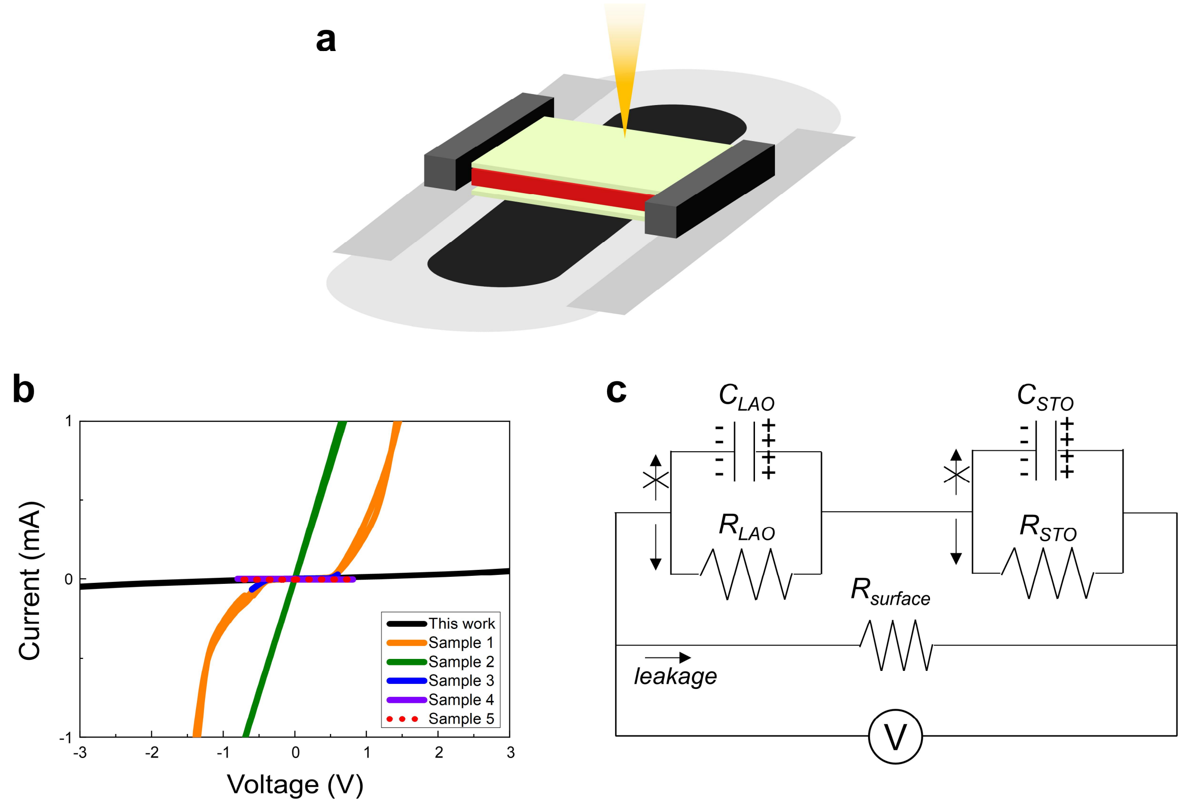

Supplementary Fig. S6. I-V characteristics of TEM samples of the $LaAlO_3/SrTiO_3$ heterostructure devices. a, Schematic illustration of a TEM sample attached to MEMS chip. Electrically dead layers are likely to form on the surfaces of the TEM sample due to FIB damages. **b,** I-V characteristics of TEM samples tested in this study. With the varying extent of FIB damages, TEM samples exhibit various I-V characteristics. The TEM sample which exhibits the lowest current level (black curve) was selected for the in-depth electrical biasing experiment. **c,** Electrical circuitry proposed for the $LaAlO_3/SrTiO_3$ heterostructure region of the TEM sample. We envision that each layer in the $LaAlO_3/SrTiO_3$ heterostructure region is a parallel combination of capacitor and resistor. The surface layers caused by FIB damages can be treated as a resistor which is in parallel connection with the entire $LaAlO_3/SrTiO_3$ heterostructure region.

	Mean inner potential, V_0 (V)	
	Charged atom model	Neutral atom model
$SrRuO_3$	16.67[1]	23.82[1]
$LaAlO_3$	17.29[1]	25.95[1] 25.76[2]
$SrTiO_3$	15.10[1]	22.40[1] 22.25[2]

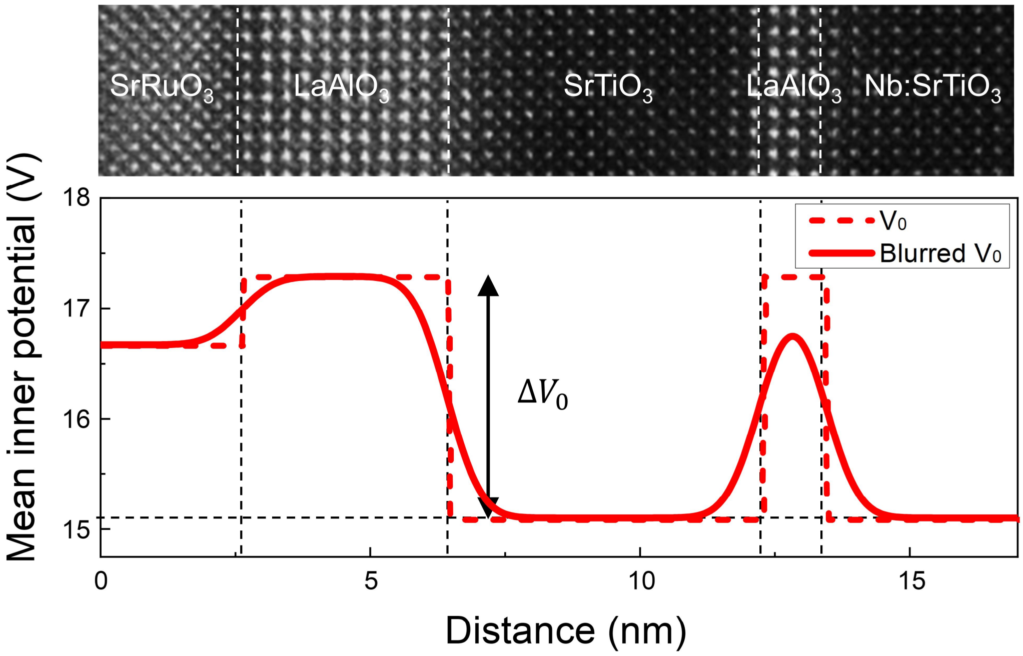

Supplementary Fig. S7. Mean inner potential (V_0) profile across the $LaAlO_3/SrTiO_3$ heterostructure device. Summary of the mean inner potential (V_0) of each layer calculated by using the atomic scattering factors of charged and neutral atoms. The calculated V_0 based on charged atom model is plotted across each layer in the heterostructure (dashed line). We note that the V_0 difference between the layers (ΔV_0) dominates the phase signal of inline electron holography results. To take account of a finite resolution of inline electron holography, the V_0 profile is blurred by a point spread function (solid line).

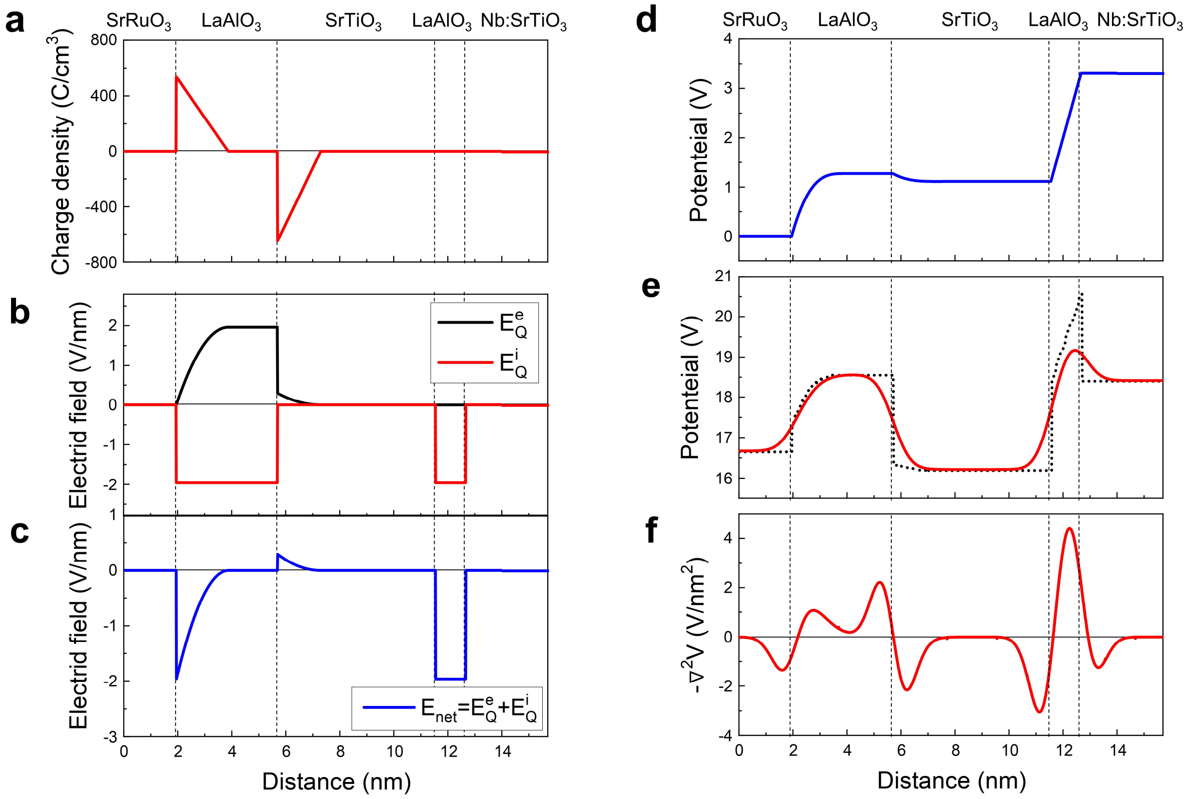

Supplementary Fig. S8. Charge density profile derived from a charge model for the $LaAlO_3/SrTiO_3$ heterostructure. a, Charge model built upon the interface charges which are oxygen vacancy and 2DEG present near the $LaAlO_3$ surface and the $LaAlO_3/SrTiO_3$ interface, respectively. **b,** Electric field (E_Q^e) calculated from the charge model (black line) and the intrinsic polar field (E_Q^i) originating from polar discontinuity (red line). **c,** Net electric field (E_{net}) profile obtained by summation of E_Q^e and E_Q^i. **d,** Potential profile calculated from the net electric field. **e,** Measurable net potential by electron holography, which includes the potential from the net electric field and mean inner potential (black dash line). The net potential is blurred by a point spread function to take account of the finite resolution of the inline electron holography technique. **f,** Inverse Laplacian of the net potential curve which is equivalent to the charge density profile measured by inline electron holography. The ΔV_0 across the interfaces introduces a pair of peaks with opposite signs.

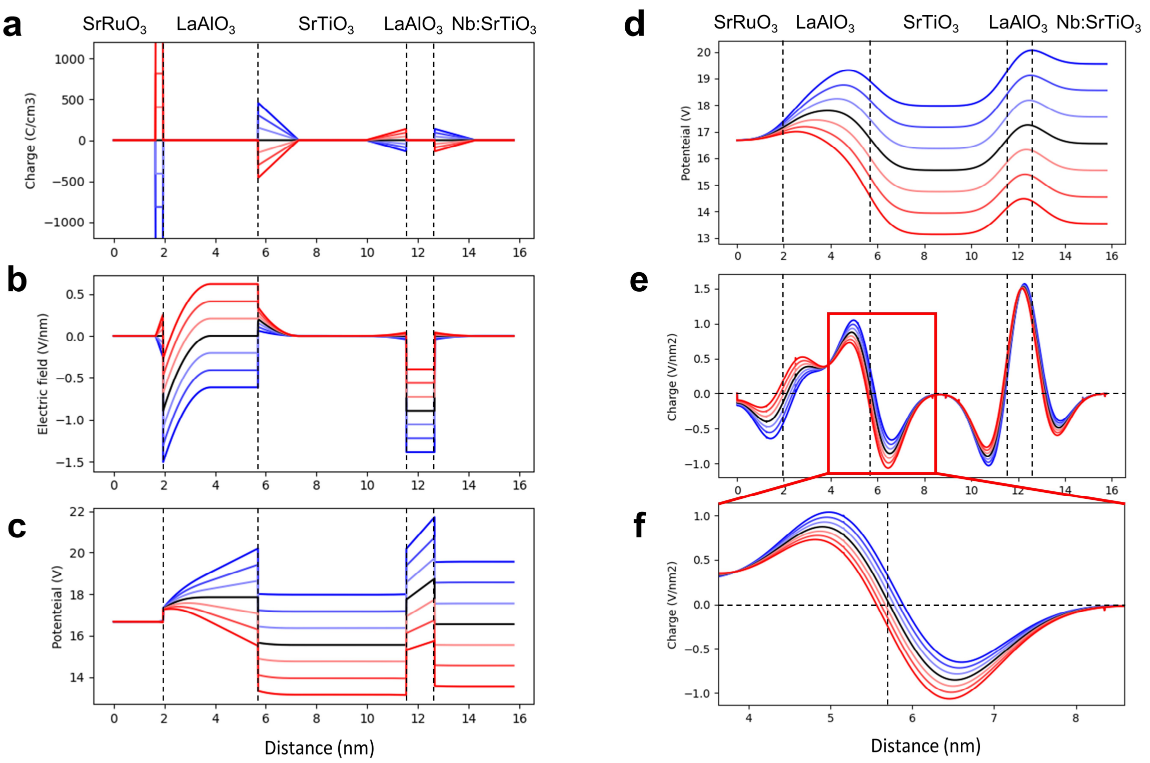

Supplementary Fig. S9. Change of charge density profiles modeled by modulation of 2DEG density under applied votages. a, Model illustrating the modulation of interface charges induced by applied voltages. **b,** Electric field (E_{net}) profiles related to the modeled interface charges (E_Q^e). Note that the electric field includes the intrinsic polar field (E_Q^i) originating from polar discontinuity. **c,** Potential profiles calculated from the electric fields. The mean inner potential has been added to each layer to yield the net potentials. **d,** Gaussian blurred potential profiles. The net potential is blurred by a point spread function to take account of the finite resolution (~0.7 nm) of the inline electron holography technique. **e,** Charge density profiles obtained by inverse Laplacian of the (Gaussian blurred) net potential curve. **f,** Close-up of the profiles at the $LaAlO_3$/$SrTiO_3$ interface region. The profiles perfectly reproduce the experimentally measured charge density profiles. This model-based approach is suited well for the interpretation of the experimental electron holography results. To this end, we are able to conclude that the 2DEG modulation within the $SrTiO_3$ side of the interface results in the change of charge profiles extended across the interface due to a finite resolution of inline electron holography.

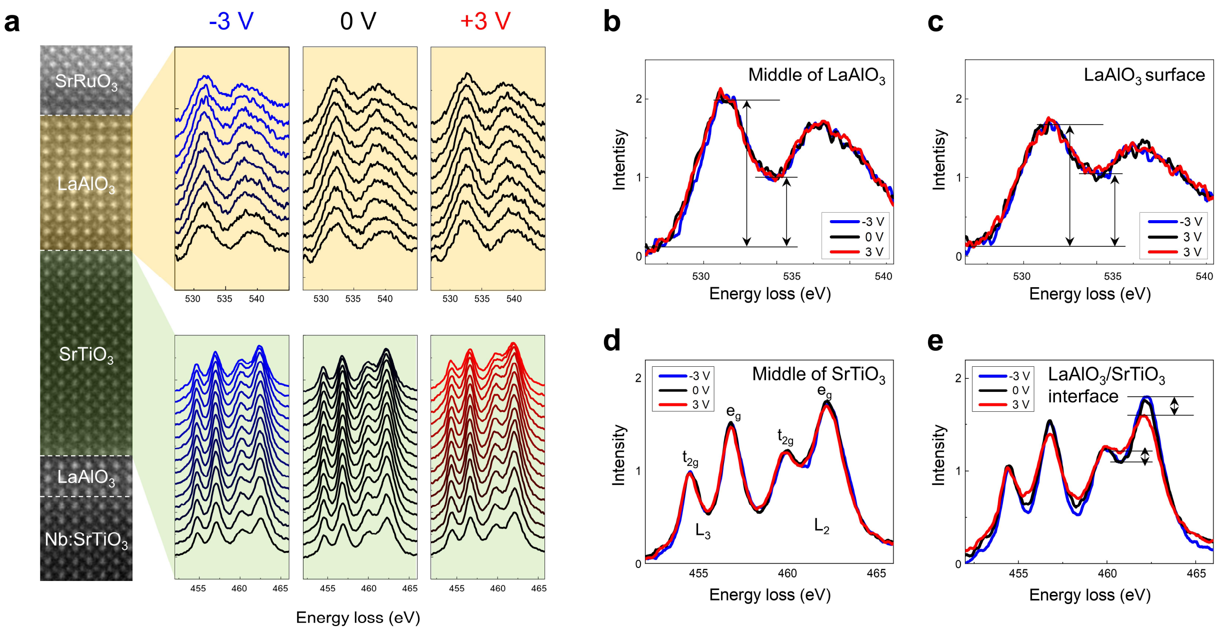

Supplementary Fig. S10. In-situ STEM EELS results and the key features in the fine structure for characterization of oxygen vacancy (V_O) and 2DEG. a, In-situ STEM EELS data of O-K and Ti-$L_{2,3}$ edges obtained at –3V, 0 V, and +3 V. The O-K edge and Ti- $L_{2,3}$ edge were collected from the $LaAlO_3$ and the $SrTiO_3$ to trace the migration of V_O and determine the valence state of Ti, respectively. EELS O-K edge from **b,** the middle of $LaAlO_3$, and **c,** the $LaAlO_3$ surface. The EEL spectra from the middle and surface of $LaAlO_3$ show a clear difference in terms of the ratio between 1st peak and the valley, which is sensitive to V_O. The reduced intensity ratio in the $LaAlO_3$ surface indicates the presence of V_O. Under the applied voltages, however, the O-K edge remains almost the same in the entire region of $LaAlO_3$. EELS Ti-$L_{2,3}$ edges from **d,** the middle of $SrTiO_3$, and **e,** the $LaAlO_3/SrTiO_3$ interface. The variation of the fine structure of Ti-$L_{2,3}$ edges supports the modulation of 2DEG near the $LaAlO_3/SrTiO_3$ interface. As the Ti^{3+} state increases with the occupation of Ti 3*d* orbitals by 2DEG, the relative intensity of the e_g peak decreases, and the valley between t_{2g} and e_g peaks increases. This trend is clearly observed under +3 V but the opposite trend under -3 V.

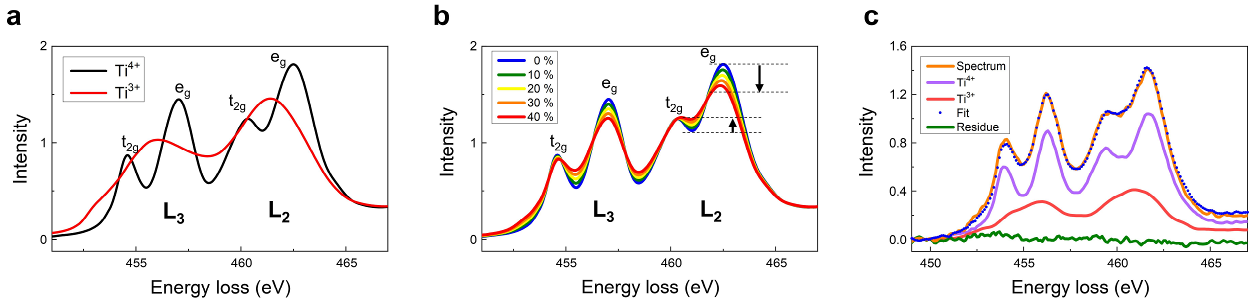

Supplementary Fig. S11. Reference spectra and multiple linear least squares (MLLS) analysis of EELS Ti-$L_{2,3}$ edge. a, Reference spectra of Ti-$L_{2,3}$ edge for Ti^{4+} (black) and Ti^{3+} (red) state acquired from $SrTiO_3$ and $LaTiO_3$, respectively. **b,** Ti-$L_{2,3}$ edge generated by weighted average of Ti^{3+} and Ti^{4+}. Arrows indicate the change of fine structure with the increase of Ti^{3+} fraction. **c,** An example of MLLS fitting to determine the Ti^{3+} fraction. The residue is used to evaluate the reliability of MLLS fitting.

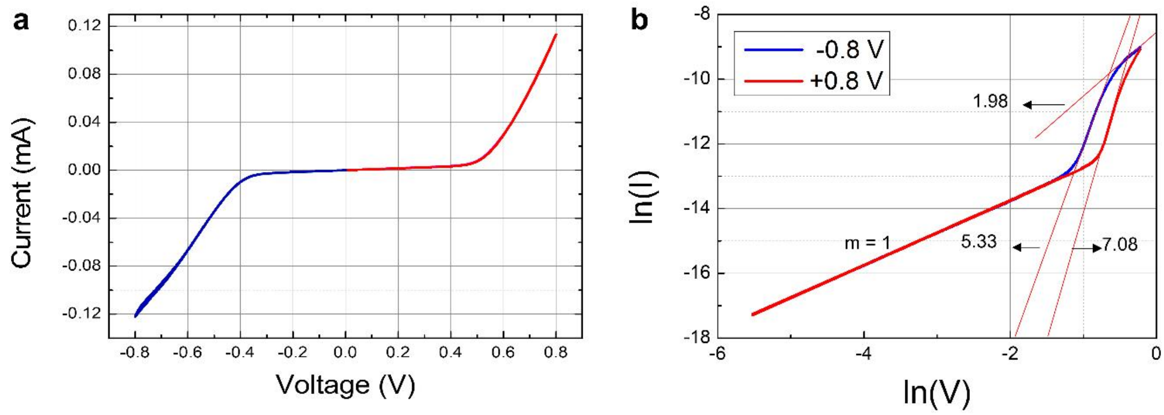

Supplementary Fig. S12. I-V curve of the $LaAlO_3$/$SrTiO_3$ heterostructure TEM sample showing the smallest leakage current (black curve in Fig. S6). a, Linear scale and **b,** log-scale plot in the voltage ranges from -0.8 to 0.8 V. The slopes (m) of three different regimes were determined by linear fitting. The transition from Ohmic (m = 1) to space-charge limited current is identified. At low voltages, the current shows a clear Ohmic (slope, m = 1) behavior, followed by transition to a trap-filling-limited (m = l+1, where l = 6.08 and 4.33 for positive and negative voltages, respectively) behaviors. At higher voltages, almost all traps are filled and thus trap-fee, space-charge-limited (m = 2) conduction behavior governs the transport.

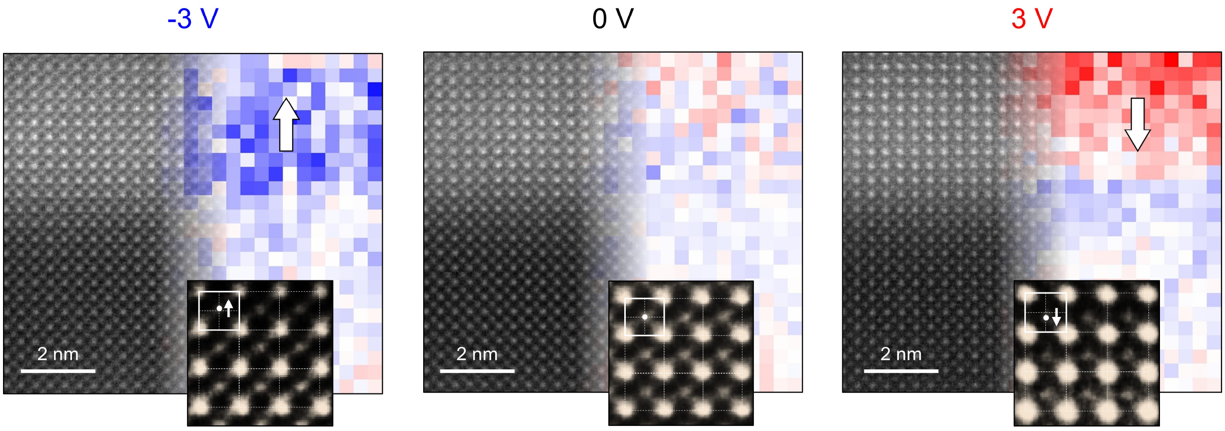

Supplementary Fig. S13. Ionic polarization determined by measuring the atomic column positions on HAADF STEM images obtained under applied voltages. Atomic-scale HAADF STEM images of the $LaAlO_3/SrTiO_3$ heterostructure acquired under -3 V, 0 V, and +3 V. The inset images show the displacement of B-site cations from the center of unit cell, which points to the same direction as the applied electric field.

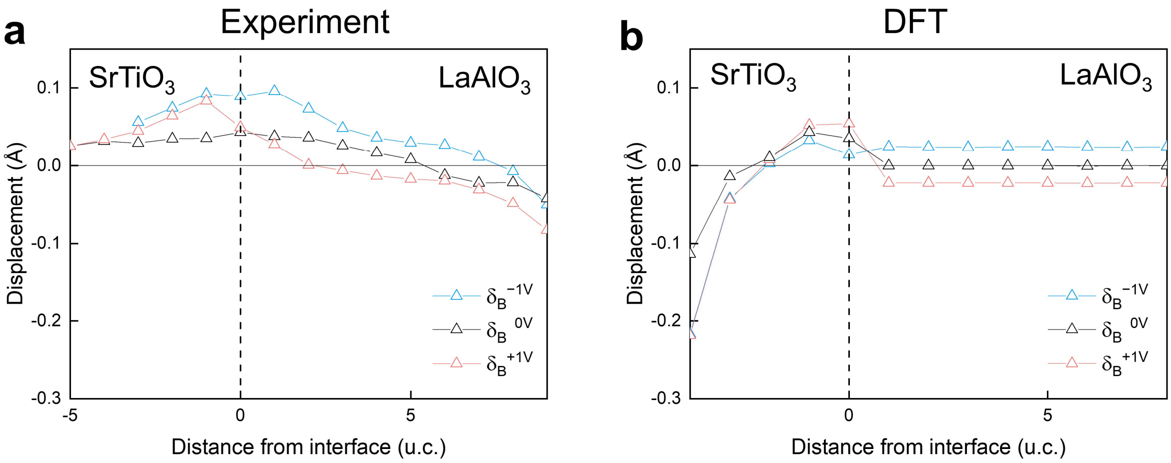

Supplementary Fig. S14. Polar distortion in the near-interface $SrTiO_3$ layers where 2DEG exists. δ_B and δ_O denotes the displacement of B-site cation and oxygen, respectively. **a,** Experimental measurement using STEM images and **b,** DFT calculation results from this study. The $SrTiO_3$ layers beneath the $LaAlO_3/SrTiO_3$ interface exhibits a noticeable polar distortion in both experiment and DFT, which is related with the accommodation of 2DEG

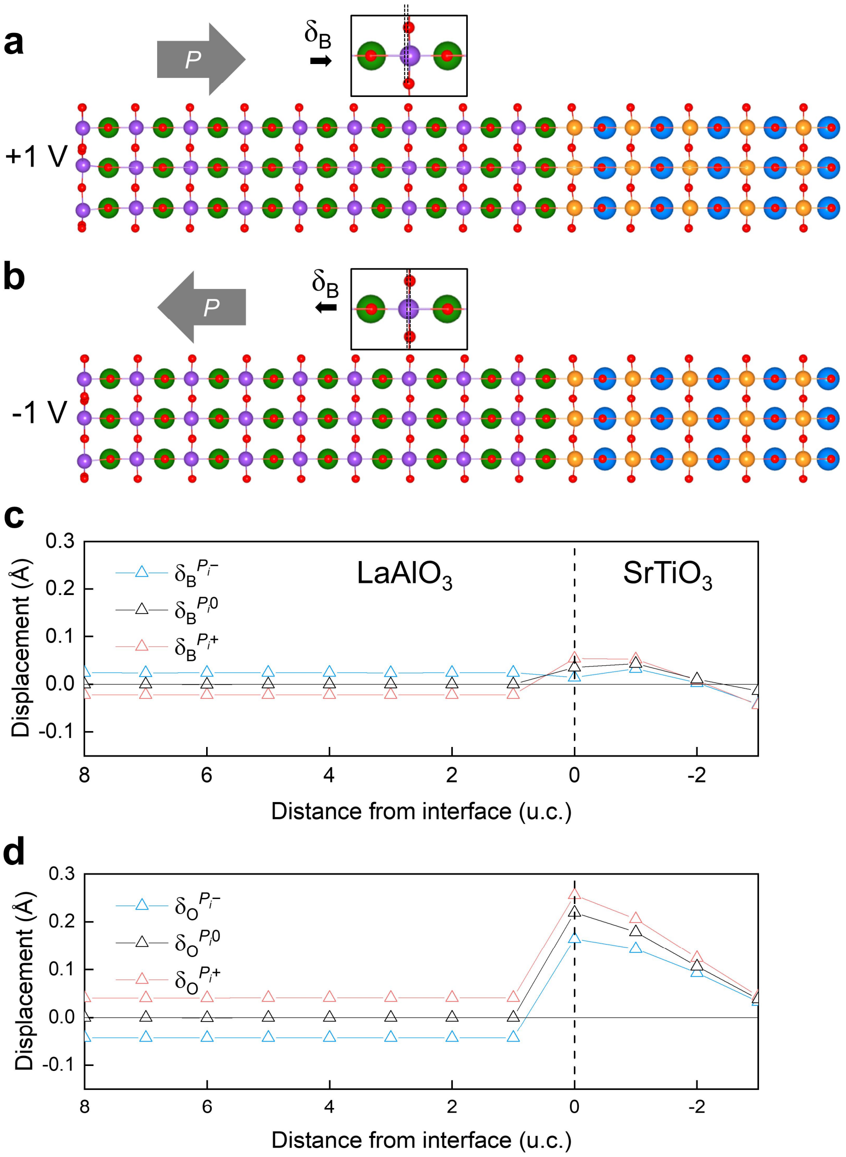

Supplementary Fig. S15. Ionic displacements of the $LaAlO_3/SrTiO_3$ slab models constructed by using experimentally measured atomic position of $LaAlO_3$. The $SrTiO_3$ layers were relaxed to monitor the modulation of 2DEG and associated polar distortion in response to the ionic polarization (P_i) in $LaAlO_3$. Structural model (2×2($LaAlO_3$)$_9$/($SrTiO_3$)$_5$ slab) with **a,** downward polarization and **b,** upward polarization in the $LaAlO_3$ layer. The atomic displacements of **c,** B-site cations (δ_B) and **d,** O atoms (δ_O) measured by DFT results. These displacements were determined by measuring the distances from center-of-mass position of A-site cations to the position of B-site cations (δ_B) or O atoms (δ_O). The positions of O atoms were averaged across the oxygen in the BO_2 plane. The $SrTiO_3$ layers beneath the $LaAlO_3/SrTiO_3$ interface exhibits a noticeable polar distortion in both experiment and DFT, which is related with the accommodation of 2DEG

References

1. Peng, L.-M. Electron scattering factors of ions and their parameterization. *Acta Crystallogr A* **54**, 481–485 (1998).
2. Doyle, P. A. t & Turner, P. S. Relativistic Hartree–Fock X-ray and electron scattering factors. *Acta Crystallogr A* **24**, 390–397 (1968).
